# Maternal folic acid supplementation and infant birthweight in low‐ and middle‐income countries: A systematic review

**DOI:** 10.1111/mcn.12895

**Published:** 2019-11-04

**Authors:** Hannah Jonker, Noa Capelle, Andrea Lanes, Shi Wu Wen, Mark Walker, Daniel J. Corsi

**Affiliations:** ^1^ Ottawa Hospital Research Institute Ottawa Ontario Canada; ^2^ Midwifery Research Centre, McMaster University Hamilton Ontario Canada; ^3^ School of Epidemiology and Public Health University of Ottawa Ottawa Ontario Canada; ^4^ Department of Obstetrics, Gynecology & Newborn Care University of Ottawa Ottawa Ontario Canada; ^5^ Children's Hospital of Eastern Ontario Research Institute Ottawa Ontario Canada

**Keywords:** birthweight, developing countries, folic acid, infant, nutritional status, pregnancy, systematic review

## Abstract

The relationship between maternal folic acid supplementation in pregnancy and infant birthweight has not been well described in low‐ and middle‐income countries. We conducted a systematic review and meta‐analysis of the current evidence of the association between folic acid supplementation in pregnancy on three primary outcomes: the incidence of low birthweight, small for gestational age, and mean birthweight. Seventeen studies were identified, which satisfied the inclusion criteria, covering a total of 275,421 women from 13 cohort studies and four randomized controlled trials. For the primary outcome of mean birthweight (*n* = 9), the pooled mean difference between folic acid and control groups was 0.37 kg (95% confidence interval [CI]: 0.24 to 0.50), and this effect was larger in the randomized controlled trials (0.56, 95% CI: 0.15 to 0.97, *n* = 3). The pooled odds ratio was 0.59 for low birthweight (95% CI: 0.47 to 0.74, *n* = 10) among folic acid supplementation versus control. The pooled odds ratio for the association with small for gestational age was 0.63 (95% CI: 0.39 to 1.01, *n* = 5). Maternal folic acid supplementation in low‐ and middle‐income countries was associated with an increased mean birthweight of infants and decreases in the incidence of low birthweight and small for gestational age.

**Key messages**

- The relationship between folic acid supplementation in pregnancy and infant birthweight in low‐ and middle‐income countries is not clear.
- We find a positive association between folic acid supplementation and infant birthweight and an inverse association with the incidence of low birthweight and small for gestational age.
- Although included studies were primarily observational, findings were consistent in a subset of randomized controlled trials.
- Changes in policy concerning folic acid supplementation or fortification of food with folic acid in low‐income settings may have potential benefit for reducing the incidence of low birthweight.

## 1 INTRODUCTION

Low birthweight (LBW) is a significant public health issue in low‐ and middle‐income counties. Poor nutrition before and during pregnancy is recognized as an important cause of LBW. Two main causes of LBW are preterm birth (before 37 weeks of gestation) and intrauterine growth restriction (World Health Organization, & Unicef, 2004). Multiple pregnancies, infections, and chronic diseases can also contribute to LBW (World Health Organization, 2014). Birthweight is also affected by the mother's own fetal development and diet throughout her life, and upon pregnancy, the mother's nutrition and health play an important role in birthweight (ACC/SCN, 2000). In high‐income countries, LBW primarily occurs due to preterm birth; however, in low‐ and middle‐income countries, the cause is primarily intrauterine growth restriction (Wardlaw, 2004).

A concurrent measure to LBW is small for gestational age (SGA), defined as infants whose weight is less than the 10th percentile for gestational age (University of Rochester Medical Center, 2017). The causes of SGA may include relative placental insufficiency caused by multiple gestation, placental insufficiency, chronic maternal hypoxaemia caused by pulmonary or cardiac disease, maternal malnutrition, and conception using assisted reproductive technology (Stavis, 2017). SGA infants have similar outcomes as LBW infants; however, LBW is a more commonly used measure, as it is broader than SGA and more easily captured when the gestational age is not known.

According to the World Health Organization, 96.5% of all LBW births occur in low‐ and middle‐income countries (World Health Organization & Unicef, 2004). LBW is often used as an indicator for the mother's health and nutritional status, as well as the infant's risk of mortality and morbidity, chances of survival, long‐term health, and psychosocial development (World Health Organization, 2014).

For at least two decades, folate has been known to be associated with a reduction in pregnancy complications including neural tube defects, congenital malformations, haemorrhage, pre‐eclampsia, spontaneous abortions, and fetal growth restriction (Ramakrishnan, Manjrekar, Rivera, Gonzáles‐Cossío, & Martorell, 1999). In the human body, folate is required for the synthesis of pyrimidines and purines and the synthesis of DNA; therefore, in situations where there is rapid dividing of cells, such as in fetal development, a lack of folate may lead to alterations in DNA synthesis (Ramakrishnan et al., 1999). With this knowledge, it is clear that folate plays a critical role in fetal growth and development, and therefore, the maternal folate status can play an important role in the development of a variety of problems for the fetus. Despite the large amount of evidence linking poor maternal folate status to the development of neural tube defects, there has been relatively limited number of investigations into the association between low maternal folic acid (FA) supplementation and incidence of LBW.

The objective of this study was to examine the effects of FA supplementation in pregnant women on the birthweight of infants and the incidence of LBW and SGA in low‐ and middle‐income countries. We sought to synthesize all available literature and produce pooled estimates of the association between FA supplementation and birthweight and prevalence of LBW/SGA.

## 2 METHODS

The study population was pregnant women and their infants in low‐ and middle‐income countries, and the intervention was FA supplementation. The comparison was the absence of dietary FA supplementation, and the outcome was association between FA supplementation and LBW, SGA, and mean birthweight. For comparability across studies, effect estimates were calculated by the authors using extracted means and counts of events. The study design is a systematic review of studies published between 1990 and 2017.

### 2.1 Data sources

Two databases were used: Ovid Embase (1974–2017) and Ovid Medline (including Epub Ahead of Print, In‐Process & Other Non‐Indexed Citations, Ovid MEDLINE® Daily and Ovid MEDLINE®, 1946 to present). Keywords for folate included folate and folic acid. Low‐ and middle‐income countries were selected using a definition from the UN World Economic Situation and Prospects 2016 (United Nations, 2016) including low‐income countries, lower middle‐income countries, least developed countries, small island developing countries, and landlocked developing countries. The full search strategy appears in Appendix A.

The search was limited to studies from 1990–present to align with the increased use of FA beginning in the 1990s. The search was limited studies in English or French. The search strategy was created by the authors and was reviewed and augmented by a professional medical librarian.

### 2.2 Study selection

The systematic review program, Covidence, was used to manage all the studies produced by the search strategy. Two assessors independently reviewed the titles, abstracts, and full text articles, and differences in opinion were discussed until consensus was reached. Included studies covered pregnant women in low‐ and middle‐income countries who used FA supplements, FA with iron supplements (IFA), or where dietary folate or serum/blood folate levels were captured. We compared women with FA use and those receiving higher levels of dietary folate intake to those who did not take supplements or who had low levels of dietary folate. Studies were excluded if the population was outside of low‐ and middle‐income countries, no comparison group was defined, if women were only taking multivitamin supplements without indication of FA, or if FA was combined with another vitamin other than iron.

All studies identified through systematic searching were first screened against inclusion criteria based on titles and abstracts. Following title and abstract screening, remaining studies were evaluated based on a full text screening using the same inclusion/exclusion criteria. Studies that did not fit the inclusion criteria were excluded, and the exclusion reason was noted for each study.

### 2.3 Data extraction

The primary reviewer (H. J.) conducted data extraction. The following data were extracted from the studies: study title, last name of first author, date of publication, journal, contact information, funding source, industry involvement, type of publication, study design, country(ies) of study, study population, size of population, time period of the study, study objective, form of FA given, frequency of FA, how supplement use was verified, how birthweight was measured, definition of LBW, what births were excluded, mean birthweights for study and control groups, counts and percentage LBW or SGA for study and control groups, and any major limitations.

### 2.4 Risk of bias assessment

All included studies were evaluated for risk of bias. Nonrandomized cohort and case–control studies were evaluated for risk of bias using the Newcastle–Ottawa Quality Assessment Scale (Wells et al., 2017). Nonrandomized studies were awarded stars for control of bias across the following domains of study design and quality of reporting: study methods for selection, comparability, and exposure ascertainment (Table 1). More stars indicate less risk of bias. Randomized controlled trials were evaluated using the Cochrane risk‐of‐bias tool for randomized trials (Higgins & Altman, 2008) and assessed for selection bias, performance bias, detection bias, attrition bias, and reporting bias. Studies were assessed as low, unclear, or high risk of bias across domains.

**Table 1:** Characteristics of studies included in review

Author and year of publication	Country	Design	Study population size	Form of FA	Outcome variables	Risk of bias score
Abdullahi et al., 2014	Sudan	Cohort: cross‐sectional	856	IFA or FA supplements	LBW and mean BW	6 stars
Achadi et al., 1995	Indonesia	Cohort: cross‐sectional	451	IFA supplements	Mean BW	7 stars
Amuna et al., 2012	South Africa	Randomized controlled trial	120	Daily diet and formulated food multimix	Mean BW	Unclear
Balarajan et al., 2013	India	Cohort	22,648	IFA supplements	LBW	7 stars
Bawadi et al., 2010	Jordan	Cohort	700	Any form of supplementation	Mean BW	8 stars
Chaudhary et al., 2012	India	Cohort	290	IFA supplements	LBW and mean BW	7 stars
Christian et al., 2003	Nepal	Randomized controlled trial	4,926	FA supplements	LBW, SGA and mean BW	Low risk
Dwarkanath et al., 2013	South India	Cohort	1,838	Dietary folate and FA supplementation	SGA	8 stars
Joseph et al., 2011	South India	Cohort	194	IFA supplementation	LBW	8 stars
Krishnaveni et al., 2014	India	Cohort	656	Serum folate and FA supplementation	Mean BW	9 stars
Nisar 2014	Pakistan	Cohort	5,692	IFA supplements	LBW and SGA	7 stars
Ndyomugyenyi & Magnussen, 2000	Uganda	Randomized controlled trial	860	IFA supplementation	Mean BW	Low risk
Passerini et al., 2012	Vietnam	Randomized controlled trial	463	IFA supplementation	LBW	Low risk
Rao et al., 2001	India	Cohort	797	Serum folate and FA supplementation	Mean BW	8 stars
Roudbari et al., 2007	Iran	Cohort: cross‐sectional	1,109	Any form of supplementation	LBW	6 stars
Wang et al., 2016	China	Cohort	2,644	FA supplementation	LBW, SGA and Mean BW	8 stars
Zheng et al., 2016	China	Cohort	231,179	FA supplementation	SGA	9 stars

Abbreviations: BW, birthweight; FA, folic acid; IFA, FA with iron supplements; LBW, low BW; SGA, small for gestational age.

### 2.5 Meta‐analysis

A meta‐analysis was conducted of the comparable data points from each study using Rev Man 5 software. The data points compared included mean birthweights for study and control groups, incidence of LBW for study and control groups, and incidence of SGA for study and control groups. Rev Man 5 software generated forest plots and funnel plots for each of these comparable data points to summarize the odds ratio and mean differences.

## 3 RESULTS

### 3.1 Study characteristics

**Figure 1:**
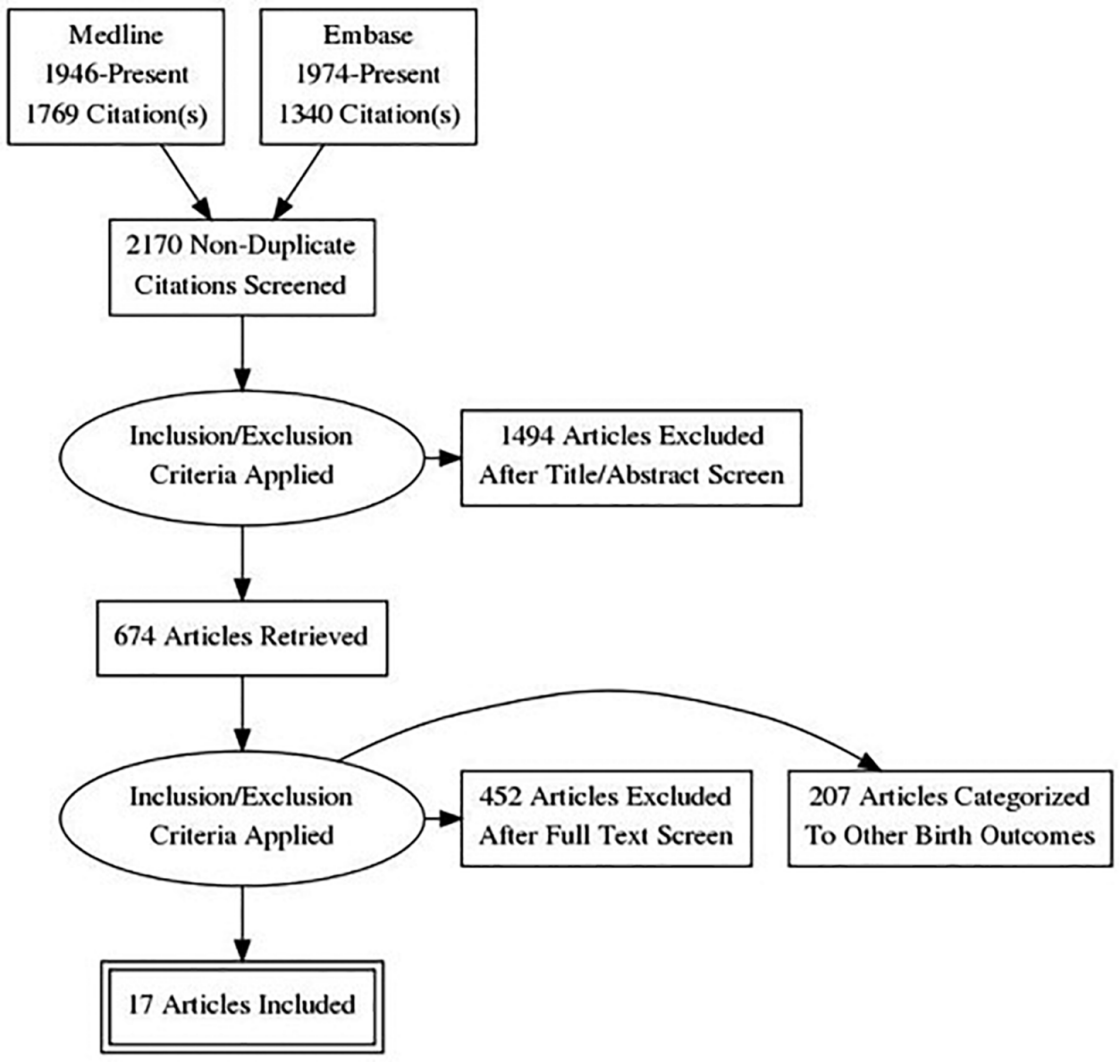
Systematic review study selection process

A total of 2,170 nonduplicated titles and abstracts were identified (Figure 1). After applying the inclusion and exclusion criteria, 674 articles were selected for full text review and assessment, which yielded 17 studies that were included in the final review and meta‐analysis. Of these, 13 were cohort studies and four were randomized controlled trials (RCTs; Table 1). Eleven studies were conducted in Asia, four in the Middle East, and two in Africa. The total combined study population was 275,421 women (min/max among included studies: 120/231,179 women). The form of FA supplementation was primarily IFA or FA supplementation, and two studies measured the blood serum folate levels instead of FA supplementation. Nine studies had the outcome variable of mean birthweight, nine had the outcome variable of LBW, and five had the outcome variable of SGA. The average risk of bias score for the cohort studies was 7.5 stars, and for the RCTs, three studies had a low risk of bias and one had an unclear risk of bias.

### 3.2 Mean birthweight

**Figure 2:**
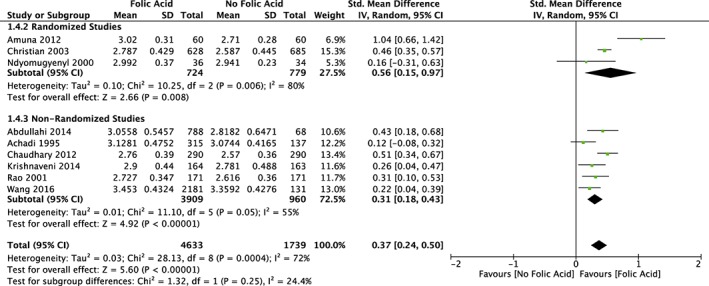
Forest plot meta‐analysis of the mean birthweight in study groups supplemented with folic acid versus control groups

**Figure 3:**
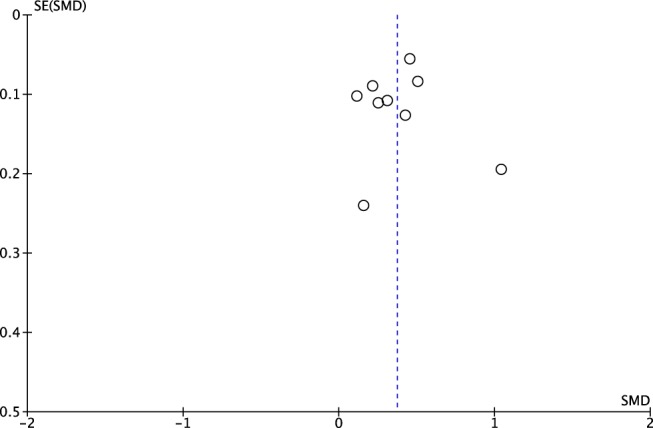
Funnel plot of the pooled mean difference for birthweights for study groups versus controls

For those studies that had outcome variables of mean birthweight, the pooled mean difference across all studies (*n* = 9) was 0.37 kg (95% CI [0.18, 0.43]) with a range of effect sizes between 0.24 and 0.50 kg (Figure 2). There was statistical heterogeneity between the studies (*τ* ^2^ = 0.03; *χ* ^2^ = 28.13, *df* = 8, *P* = .00004; *I* ^2^ = 72%). Among a subset of RCTs (*n* = 3), the pooled mean difference was 0.56 kg (95% CI [0.15, 0.97]). Individually, all studies had a statistically significant mean difference in birthweights by FA supplementation. The funnel plot for the mean difference between birthweights for all the studies demonstrates a fairly symmetric distribution of the data, which demonstrates low bias in the results, even considering that there was a small amount of studies compiled (Figure 3).

### 3.3 Low birthweight

**Figure 4:**
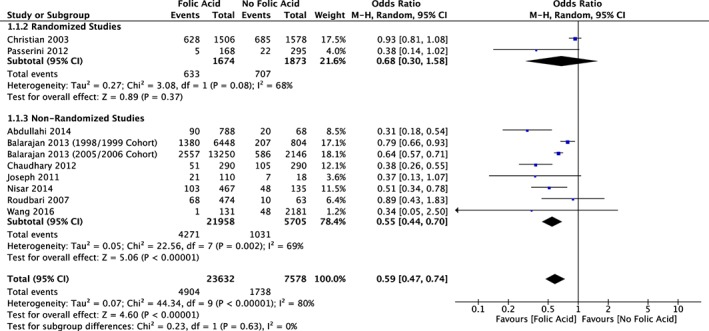
Forest plot meta‐analysis of the incidence of low birthweight in study groups supplemented with folic acid versus control groups

**Figure 5:**
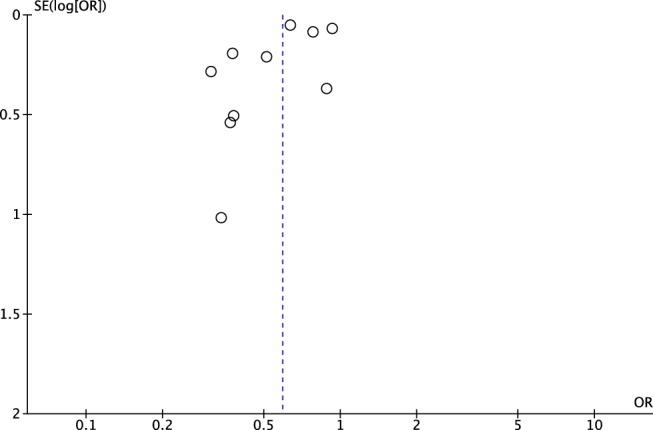
Funnel plot of the odd ratios for low birthweight for study groups versus controls

For the studies that had outcome variables of the incidence of LBW for FA supplementation groups versus control, the overall odds ratio was 0.59 (95% CI [0.47, 0.74], *n* = 10 studies; Figure 4). All studies had an odds ratio less than 1, and there was statistical heterogeneity between the FA supplementation and control groups (*τ* ^2^ = 0.07; *χ* ^2^ = 44.34, *df* = 9, *P* < .00001; *I* ^2^ = 80%). Among a subset of two RCTs, the pooled odds ratio was 0.68 (95% CI [0.30, 1.58]), although there was less statistical precision due to smaller number of studies. The funnel plot of the odds ratios for LBW produced an asymmetrical distribution, which indicates some bias in the results (Figure 5).

### 3.4 Small for gestational age

**Figure 6:**
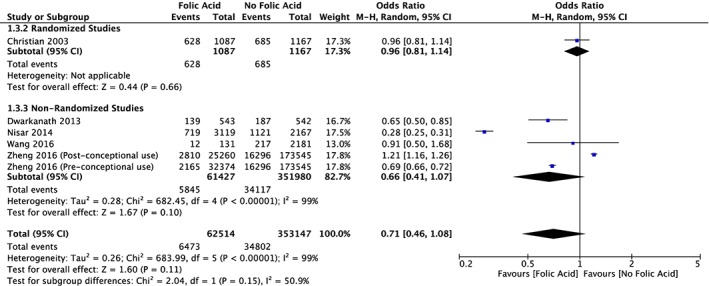
Forest plot meta‐analysis of the incidence of small for gestational age in study groups supplemented with folic acid versus control groups

**Figure 7:**
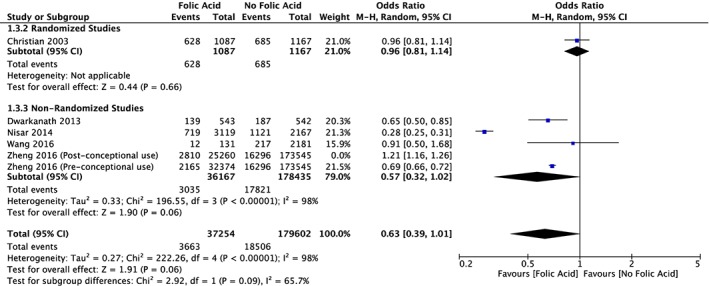
Forest plot meta‐analysis of the incidence of small for gestational age in study groups supplemented with folic acid versus control groups (Zheng, 2016, postconceptional use removed)

There were less studies with the outcome variable of SGA; however, five out of the six study groups had an odds ratio of less than 1, favouring FA supplementation (Figure 6). The total odds ratio for all six study groups was 0.71 (95% CI [0.46, 1.08]). One study group that used postconceptional FA supplementation later in the pregnancy had an odds ratio greater than 1, favouring no supplementation; however, because this form of supplementation was different from the rest, a second forest plot was created without this study group (Figure 7). The total odds ratio for this forest plot was 0.63 (95% CI [0.39, 1.01]). There were insufficient studies for this outcome variable to create an accurate funnel plot.

## 4 DISCUSSION

In this systematic review and meta‐analyses, we determined that maternal FA supplementation in low‐ and middle‐income countries was associated with an increase in the mean birthweight of infants and a decrease in the incidence of LBW and SGA. The included studies were determined to be of high quality, and stratification by randomized and nonrandomized design indicated consistency in the direction and magnitude of association. Analyses of mean birthweight demonstrated a statistically significant difference between FA and control suggesting that maternal FA supplementation can potentially increase the mean birthweight of infants in these settings.

Analyses of LBW demonstrated statistically significant heterogeneity in the incidence of LBW between FA supplementation and control groups, and the odds ratios overall and for all studies individually were in favour of FA supplementation. The funnel plot for the LBW odds ratios demonstrated a less symmetric distribution of the data points, which points to some bias in the results; however, it is possible that with a greater number of studies to compare this distribution would become more symmetrical.

The SGA forest plot also demonstrated a statistically significant heterogeneity between the incidence of SGA in the FA supplementation and control groups. One study group was removed from this forest plot due to the fact that postconception FA supplementation was used and it was a different form of supplementation than the rest of the studies compared (Zheng et al., 2016). After this study was removed, the total odds ratio decreased even further, favouring FA supplementation (Figure 7). The total odds ratio for SGA was higher than the total odds ratio for LBW, and this is probably partially due to the fact that fewer studies had the outcome variable of SGA.

### 4.1 Comparison with other studies

To our knowledge, this is the first comprehensive systematic review and meta‐analysis on the effect of maternal FA supplementation on infant birthweight in low‐ and middle‐income countries. The existing literature is composed of nonsystematic reviews without published search strategies or meta‐analyses. One study that has been published has shown a positive association between FA supplementation and birthweight (Nguyen et al., 2012). Another article from 2009 indicated that blood serum folate levels were positively associated with birthweight and that IFA supplements increased birthweight (Muthayya, 2009). Another review article conducted in 2012 also found that IFA supplementation may have an association on the incidence of LBW in Ethiopia, India, and Nigeria (Mason et al., 2012). These findings have important implications for low‐ and middle‐income countries where FA deprivation may continue over successive generations of mothers and contribute to intergenerational cycle of LBW infants.

### 4.2 Strengths and limitations

Looking beyond predominantly larger RCTs, which mostly occur in high‐income countries, this review included observational and RCTs conducted in low‐ and middle‐income countries. The predominance of observational data may have increased the potential for risk of bias or confounding; however, it was of particular interest to examine the situation in low‐ and middle‐income countries where the greatest burden exists and greatest potential for benefit. The socio‐economic context of low‐ and middle‐income countries vary considerably from high‐income countries, and in order to generate evidence to inform policy in these contexts, the review was restricted to low‐ and middle‐income countries. Another strength of this study is that risk of bias was individually assessed for all studies included in the analysis and all cohort studies had above a six‐star score and three out of four RCTs had a low risk of bias. This study also used a reproducible method that could be used to re‐examine the literature in the future. Taken together and using the GRADE criteria for certainty in the level of evidence, we believe that the underlying effect is probably close to our pooled estimates (moderate certainty).

The limitations of this systematic review include the fact that LBW is a very complicated issue with many multidimensional factors that contribute to its aetiology, including maternal nutrition, socio‐economic status, chronic diseases, infectious diseases, pollution, preterm birth, and genetics. We only considered FA supplementation as a single aspect of maternal nutrition. Another confounding factor in this study is that many of the studies included in the review used IFA supplementation, so the effects of the supplementation with FA cannot be entirely separated from iron supplementation. We chose to include these studies despite this concern due of the limited number of studies, and in low‐income settings, FA is typically combined with iron in a single supplement. As with all reviews, there is the possibility that publication bias may have skewed the direction of association in the published literature. Another confounding factor may be the patterning of the use of FA supplements by socio‐economic status and other maternal characteristics that may have not been completely controlled for in the observational studies. Due to heterogeneity across studies in the number and types of covariate‐adjusted analyses, we pooled only the unadjusted effect estimates. Although these estimates may be subject to residual confounding in the observational studies, there was a consistency in the effect estimates between the randomized and nonrandomized studies. Future research is needed to evaluate the other factors contributing to LBW in low‐ and middle‐income countries in order to find interventions to decrease its incidence.

## 5 CONCLUSIONS AND IMPLICATIONS

In conclusion, maternal FA supplementation had a statistically significant and positive association on birthweight and an inverse association with incidence of LBW and SGA in low‐ and middle‐income countries. FA supplementation is already widely recommended for the prevention of neural tube defects; however, changes in policy concerning FA supplementation or fortification of food with FA has lagged behind in low‐ and middle‐income countries. These results and potential benefit of FA supplementation can contribute to simple low‐cost interventions aimed at reducing the incidence of LBW worldwide.

## CONFLICTS OF INTEREST

The authors declare that they have no conflicts of interest.

## PROTOCOL REGISTRATION

The systematic review protocol was registered on Prospero (CRD42017068273).

## CONTRIBUTIONS

HJ, MW, and DC conceived and designed the study. HJ and NC performed the review. HJ analysed the data and drafted the manuscript. All authors participated in interpretation of the data and critical revisions of the manuscript.

## APPENDIX A 1

### SEARCH STRATEGY

The search strategy included the following keyword and Medical Subject Heading search terms: folic acid, folate, drug administration, drug combination, drug therapy, oral drug administration, therapy, supplement, diet, pill, oral, therapy, pregnancy, pregnancy outcome, pregnancy complication, perinatal care, prenatal care, perinatal mortality, perinatal morbidity, maternal mortality, maternal welfare, maternal, perinatal, prenatal, pregnant, developing country, low‐income country, developing nation, least developed, less developed, third world, under developed, and low income or middle income. In addition to these keywords, a list of low‐income countries, lower middle‐income countries, least developed countries, small island developing countries, and landlocked developing countries was amalgamated from the UN World Economic Situation and Prospects 2016 document and these country names were added to the search strategy (United Nations, 2016).

## APPENDIX B 1

### RISK OF BIAS ASESSMENT

**Table B1:** Risk of bias assessment for randomized controlled trials, Cochrane risk‐of‐bias tool

Author	Date	Adequate generation of allocation sequence	Allocation concealment	Blinding of participants and personnel	Blinding of outcome assessors	Incomplete outcome data	Selective outcome reporting	Other sources of bias	Total risk of bias
Amuna	2012	Yes	Unclear	Unclear	Unclear	No	No	No	Unclear
Christian	2003	Yes	Yes	Yes	Yes	No	No	No	Low
Ndyomugyenyi	2000	Yes	Yes	Unclear	Unclear	No	No	No	Low
Passerini	2012	Yes	Yes	No	Yes	No	No	No	Low

**Table B2:** Risk of bias and quality assessment for nonrandomized Newcastle–Ottawa Scale

Author	Representativeness of the exposed cohort	Selection of the nonexposed cohort	Ascertainment of exposure	Outcome of interest not present at the start of the study	Comparability of cohorts on the basis of design or analysis	Assessment of outcomes	Follow‐up long enough for outcome to occur	Adequacy of follow‐up	Total stars
Abdullahi	Representative of the average pregnant women in developing countries^*^	Drawn from the same community as the exposed cohort^*^	Self‐report	Yes^*^	Both^**^	Self‐report	Yes^*^	No statement	6
Achadi	Representative of the average pregnant women in developing countries^*^	Drawn from a different source	Self‐report	Yes^*^	Both^**^	Record linkage^*^	Yes^*^	Complete follow‐up—all subjects accounted for^*^	7
Balarajan	Representative of the average pregnant women in developing countries^*^	Drawn from the same community as the exposed cohort^*^	Self‐report	Yes^*^	Both^**^	Self‐report	Yes^*^	Complete follow‐up—all subjects accounted for^*^	7
Bawadi	Representative of the average pregnant women in developing countries^*^	Drawn from the same community as the exposed cohort^*^	Self‐report	Yes^*^	Both^**^	Record linkage^*^	Yes^*^	Complete follow‐up—all subjects accounted for^*^	8
Nisar	Representative of the average pregnant women in developing countries^*^	Drawn from the same community as the exposed cohort^*^	Self‐report	Yes^*^	Both^**^	Self‐report	Yes^*^	Subjects lost to follow‐up unlikely to introduce bias—small number lost (i.e., >90% followed up or description provided of those lost not indicative of a difference in attrition between exposed and nonexposed groups)^*^	7
Chaudhary	Representative of the average pregnant women in developing countries^*^	Drawn from the same community as the exposed cohort^*^	Record linkage^*^	Yes^*^		Record linkage^*^	Yes^*^	Complete follow‐up—all subjects accounted for^*^	7
Dwarkanath	Representative of the average pregnant women in developing countries^*^	Drawn from the same community as the exposed cohort^*^	Self‐report	Yes^*^	Both^**^	Record linkage^*^	Yes^*^	Subjects lost to follow‐up unlikely to introduce bias—small number lost (i.e., >90% followed up or description provided of those lost not indicative of a difference in attrition between exposed and nonexposed groups)^*^	8
Joseph	Representative of the average pregnant women in developing countries^*^	Drawn from the same community as the exposed cohort^*^	Self‐report	Yes^*^	Both^**^	Record linkage^*^	Yes^*^	Subjects lost to follow‐up unlikely to introduce bias—small number lost (i.e., >90% followed up or description provided of those lost not indicative of a difference in attrition between exposed and nonexposed groups)^*^	8
Krishnaveni	Representative of the average pregnant women in developing countries^*^	Drawn from the same community as the exposed cohort^*^	Record linkage^*^	Yes^*^	Both^**^	Record linkage^*^	Yes^*^	Subjects lost to follow‐up unlikely to introduce bias—small number lost (i.e., >90% followed up or description provided of those lost not indicative of a difference in attrition between exposed and nonexposed groups)^*^	9
Rao	Representative of the average pregnant women in developing countries^*^	Drawn from the same community as the exposed cohort^*^	Self‐report	Yes^*^	Both^**^	Record linkage^*^	Yes^*^	Complete follow‐up—all subjects accounted for^*^	8
Roudbari	Representative of the average pregnant women in developing countries^*^	Drawn from the same community as the exposed cohort^*^	Self‐report	Yes^*^	Both^**^	Self‐report	Yes^*^	No statement	6
Wang	Representative of the average pregnant women in developing countries^*^	Drawn from the same community as the exposed cohort^*^	Self‐report	Yes^*^	Both^**^	Record linkage^*^	Yes^*^	Complete follow‐up—all subjects accounted for^*^	8
Zheng	Representative of the average pregnant women in developing countries^*^	Drawn from the same community as the exposed cohort^*^	Self‐report	Yes^*^	Both^**^	Record linkage^*^	Yes^*^	Complete follow‐up—all subjects accounted for^*^	9

*Note:* Asterisks indicate the star rating for quality.
