# CORRIGENDUM

**DOI:** 10.1111/mcn.13193

**Published:** 2021-04-13

Author Correction: Maternal folic acid supplementation and infant birthweight in low‐ and middle‐income countries: A systematic review

## Authors and affiliation

Hannah Jonker, OMNI Research Group, Ottawa Hospital Research Institute; McMaster University

Noa Capelle, OMNI Research Group, Ottawa Hospital Research Institute

Andrea Lanes, OMNI Research Group, Ottawa Hospital Research Institute

Shi Wu Wen, OMNI Research Group, Ottawa Hospital Research Institute; School of Epidemiology and Public Health, University of Ottawa

Mark Walker, OMNI Research Group, Ottawa Hospital Research Institute; Department of Obstetrics and Gynecology, University of Ottawa

Daniel J. Corsi, PhD, School of Epidemiology and Public Health and Department of Obstetrics and Gynecology, University of Ottawa

**Correction to**: Maternal & Child Nutrition, https://onlinelibrary.wiley.com/doi/full/10.1111/mcn.12895, published 4 November 2019

This article contains a transcription error in the reported mean birthweight for the folic acid group from Christian et al. (2003). The correct value is 2.587 kg. As a result of the error, the mean difference in birthweight for folic acid supplementation was overestimated in the subgroup of randomized controlled trials. The error also affected the overall pooled mean difference. The correct estimates are 0.39 kg (95% CI [−0.27, 1.05]) and 0.32 kg (95% CI [0.15, 0.49]). The correct abstract and Figure 2 appear below. We also provide a corrected Figure 3, showing the funnel plot of mean differences between birthweights for all of the studies.

**FIGURE 2:**
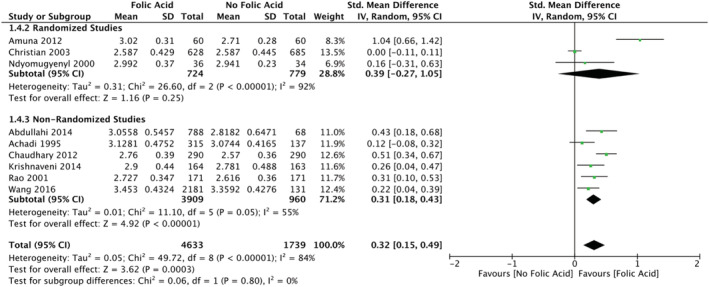
Forest plot meta‐analysis of the mean birthweight in study groups supplemented with folic acid versus control groups

**FIGURE 3:**
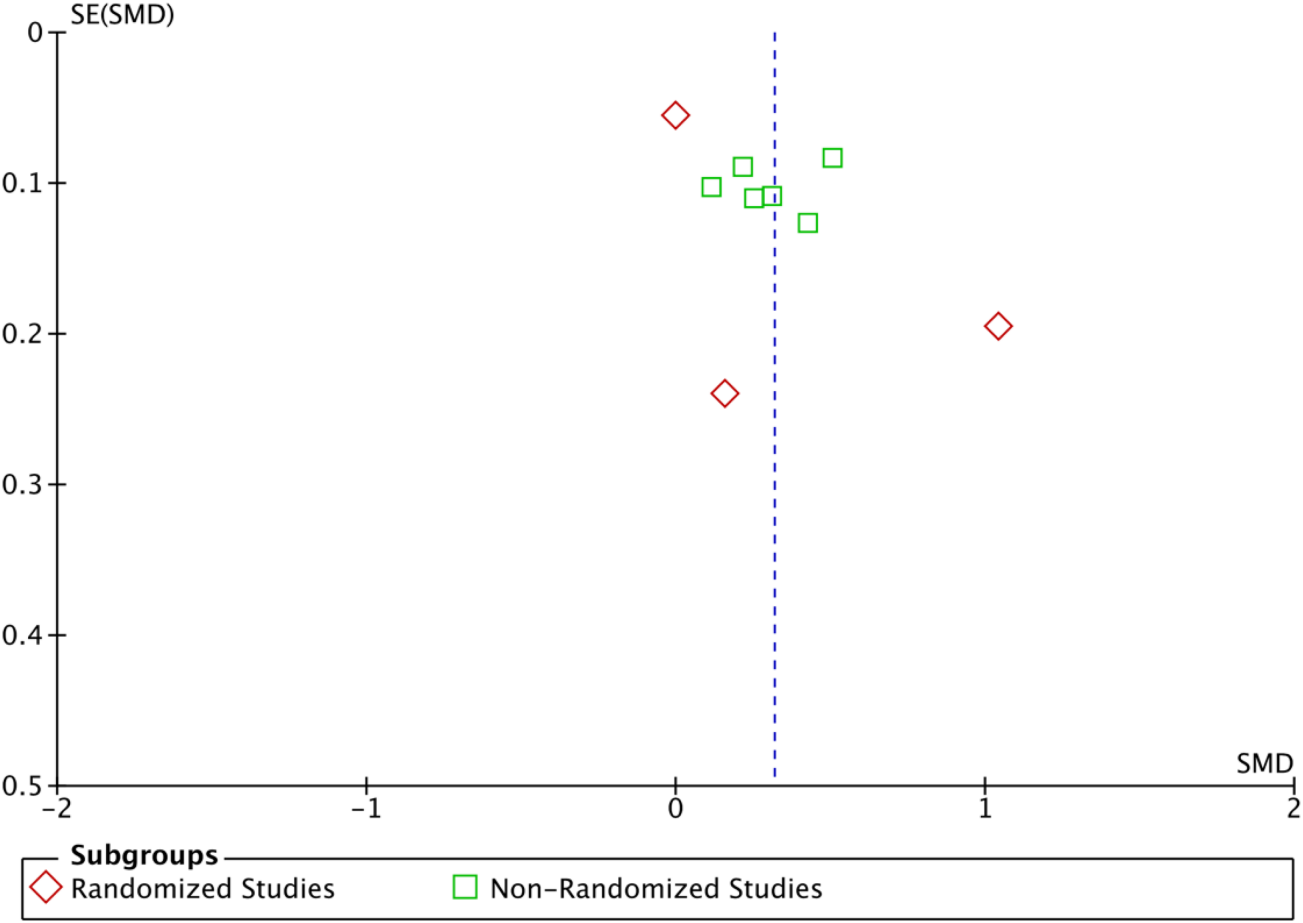
Funnel plot of the pooled mean difference for birthweights for study groups versus controls

## Abstract

The relationship between maternal folic acid supplementation in pregnancy and infant birthweight has not been well described in low‐ and middle‐income countries. We conducted a systematic review and meta‐analysis of the current evidence of the association between folic acid supplementation in pregnancy on three primary outcomes: the incidence of low birthweight, small for gestational age, and mean birthweight. Seventeen studies were identified, which satisfied the inclusion criteria, covering a total of 275,421 women from 13 cohort studies and four randomized controlled trials. For the primary outcome of mean birthweight (*n* = 9 studies), the pooled mean difference between folic acid and control groups was 0.32 kg (95% confidence interval [CI]: 0.15 to 0.49), and this effect was similar in the subset of randomized controlled trials (0.39, 95% CI: −0.27 to 1.05, *n* = 3 studies) although less statistically precise. The pooled odds ratio was 0.59 for low birthweight (95% CI: 0.47 to 0.74, *n* = 10) among folic acid supplementation versus control. The pooled odds ratio for the association with small for gestational age was 0.63 (95% CI: 0.39 to 1.01, *n* = 5). Maternal folic acid supplementation in low‐ and middle‐income countries was associated with an increased mean birthweight of infants and decreases in the incidence of low birthweight and small for gestational age.

## References

[mcn13193-bib-0001] Christian, P. , Khatry, S. K. , Katz, J. , Pradhan, E. K. , LeClerq, S. C. , Shrestha, S. R. , Adhikari, R. K. , Sommer, A. , & Keith, P. W. (2003). Effects of alternative maternal micronutrient supplements on low birth weight in rural Nepal: double blind randomised community trial. BMJ, 326(7389), 571. 10.1136/bmj.326.7389.571 12637400 PMC151518

[mcn13193-bib-0002] Jonker, H. , Capelle, N. , Lanes, A. , Wen, S. W. , Walker, M. , & Corsi, D. J. (2020). Maternal folic acid supplementation and infant birthweight in low‐ and middleincome countries: A systematic review. Maternal and Child Nutrition, 16(1), e12895. 10.1111/mcn.12895 31680411 PMC7038878

